# Brixia Chest X-ray Score, Laboratory Parameters and Vaccination Status for Prediction of Mortality in COVID-19 Hospitalized Patients

**DOI:** 10.3390/diagnostics13122122

**Published:** 2023-06-20

**Authors:** Jusuf A. Nukovic, Valentina Opancina, Nebojsa Zdravkovic, Nikola Prodanovic, Ana Pejcic, Miljan Opancina, Jasmin J. Nukovic, Radisa Vojinovic, Dragan Dulovic, Fehim Jukovic, Nedim Hamzagic, Merisa Nukovic, Nenad V. Markovic

**Affiliations:** 1 Faculty of Pharmacy and Health Travnik, University of Travnik, 72270 Travnik, Bosnia and Herzegovina; 2 Department of Radiology, General Hospital Novi Pazar, 36300 Novi Pazar, Serbia; 3 Department of Radiology, Faculty of Medical Sciences, University of Kragujevac, 34000 Kragujevac, Serbia; 4 University Clinical Center Kragujevac, 34000 Kragujevac, Serbia; 5 Department of Medical Statistics and Informatics, Faculty of Medical Sciences, University of Kragujevac, 34000 Kragujevac, Serbia; 6 Department of Surgery, Faculty of Medical Sciences, University of Kragujevac, 34000 Kragujevac, Serbia; 7 Department of Pharmacology and Toxicology, Faculty of Medical Sciences, University of Kragujevac, 34000 Kragujevac, Serbia; 8 Military Medical Academy, Faculty of Medicine, University of Defense, 11000 Belgrade, Serbia; 9 Health Center Tutin, 36320 Tutin, Serbia

**Keywords:** coronavirus disease 2019, chest radiography, laboratory, mortality

## Abstract

Chest X-ray has verified its role as a crucial tool in COVID-19 assessment due to its practicability, especially in emergency units, and Brixia score has proven as a useful tool for COVID-19 pneumonia grading. The aim of our study was to investigate correlations between main laboratory parameters, vaccination status, and Brixia score, as well as to confirm if Brixia score is a significant independent predictor of unfavorable outcome (death) in COVID-19 patients. The study was designed as a cross-sectional multicentric study. It included patients with a diagnosed COVID-19 infection who were hospitalized. This study included a total of 279 patients with a median age of 62 years. The only significant predictor of unfavorable outcome (death) was Brixia score (adjusted odds ratio 1.148, *p* = 0.022). In addition, the results of the multiple linear regression analysis (R^2^ = 0.334, F = 19.424, *p* < 0.001) have shown that male gender (B = 0.903, *p* = 0.046), severe COVID-19 (B = 1.970, *p* < 0.001), and lactate dehydrogenase (B = 0.002, *p* < 0.001) were significant positive predictors, while albumin level (B = −0.211, *p* < 0.001) was a significant negative predictor of Brixia score. Our results provide important information about factors influencing Brixia score and its usefulness in predicting the unfavorable outcome (death) of COVID-19 patients. These findings have clinical relevance, especially in epidemic circumstances.

## 1. Introduction

Severe acute respiratory syndrome coronavirus 2 (SARS-CoV-2) causing coronavirus disease 2019 (COVID-19) has had its rapid outbreak since the first patient report in late 2019, which was soon followed by the declaration of pandemic in 2020 [1,2]. Since the beginning of the pandemic, over 657 million cases and over 6.7 million deaths have been reported worldwide [3]. COVID-19 clinical manifestations have been changing over time, from respiratory infection to more of a systemic disease, which in a severe form can lead to multiple organ dysfunction, hypoxia and fatal outcome [1]. Scientifically, this period generated a vast amount of data, which is useful not only for the current pandemic, but also as a foundation for future epidemic or pandemic circumstances, especially for patients with severe clinical presentations who require critical care management and good monitoring tools.

The diagnosis of COVID-19 involves confirmation on the real-time reverse transcriptase polymerase chain reaction (RT-PCR) test, as well as on the antigen and the enzyme-linked immunosorbent assay (ELISA) tests [1]. In addition, as a standard part of the initial diagnosis of COVID-19, chest radiography (CXR) is performed primarily, as well as computed tomography (CT) of the chest [1,2,4]. CXR has verified its role as a crucial tool in COVID-19 assessment due to its practicability, especially in emergency units [5]. The basic characteristics of COVID-19 on lung radiography usually include bilateral lung opacities and an accentuated interstitial pattern [1,2]. An important role in establishing a radiological diagnosis is played by different systems for scoring the findings [1,2,4]. To this day, Brixia score has proven as a useful, clear, and straightforward tool for COVID-19 pneumonia grading, which helps clinicians to gain relevant data from CXR [6]. Multiple studies have validated Brixia score and also investigated its predictive value for patients with a diagnosis of COVID-19 pneumonia. It was found that Brixia score is associated with death and atherothrombotic complications during hospitalization and after hospital discharge [5]. Others reported that higher values of Brixia score are connected with intubation, demand for non-invasive ventilation and fatal outcome, setting a cut-off score of six as intubation predictor [7]. Further to this, it was stated that there is a significant association between the initial CXR Brixia score and its higher values with higher C-reactive protein (CRP) levels [8].

Along with the radiological examination, laboratory analysis also plays an important role in COVID-19 assessment, and its results can be a great prognostic tool for poor treatment outcome, as well as a good monitoring tool during hospital treatment. It is known that inflammatory markers (such as serum C-reactive protein, D-dimer, ferritin) rise in critically ill patients and that a large release of pro-inflammatory cytokines occurs [9,10,11,12,13]. In addition, laboratory markers such as platelets and lymphocyte count are associated with initial lung findings *p* < 0.05, *p* < 0.01, respectively [14].

Along with the clinical studies, COVID-19 pandemic related research was also developed in the field of artificial intelligence. A decision support system was created, based on machine learning and deep learning techniques, with the aim to predict the COVID-19 diagnosis using clinical, laboratory, and demographic variables [15]. Furthermore, another study created a model with 92% accuracy, which obtains a preliminary COVID-19 diagnosis, thanks to the demographic and epidemiological parameters [16]. Similar to this, a machine learning-based prediction model was created that detects COVID-19 by asking basic questions, based on symptoms [17]. These models and others are valuable sources for future epidemics/pandemics, since the use of artificial intelligence can help and ease the burden on medical infrastructure and speed up the process of diagnosis and treatment with the main goal of helping patients achieve best possible outcome.

The objective of our research was to investigate correlations between main laboratory parameters, vaccination status, and Brixia score. In addition, our aim was to investigate if Brixia score is a significant independent predictor of unfavorable outcome (death) in COVID-19 patients who were hospitalized.

## 2. Materials and Methods

**Figure 1:**
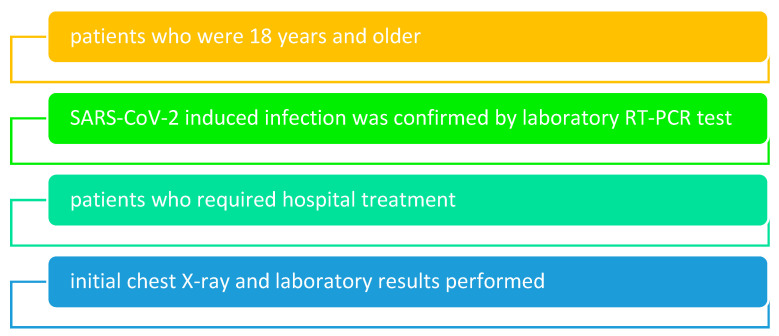
Study inclusion criteria.

The study was designed as a cross-sectional multicentric study. It included patients with a diagnosed COVID-19 infection who were admitted and treated at the University Clinical Center Kragujevac in Serbia and General Hospital Novi Pazar in Serbia, from 1 September 2021 to1 February 2022. Study inclusion criteria are presented in Figure 1. We excluded patients with incomplete documentation or artifacts on chest X-ray. The University Clinical Center in Kragujevac, Serbia, has granted ethics approval (Approval Number 01/21/240 from 26 May 2021). The Principles of Good Clinical Practice and the Helsinki Declaration were followed throughout the research process. Informed consent for the study participation was signed by patients or family member/legal representatives, upon the hospital admission.

According to the institutional COVID-19 treatment protocols at the time of the study, only patients with confirmed COVID-19 on RT-PCR test were hospitalized in COVID-19 units. All of the hospitalized patients underwent initial CXR and laboratory testing during the first six hours of hospital admission. CXR was posteroanterior or anteroposterior view, depending on the patient’s condition. The laboratory analysis set was standardized for all hospitalized patients and included blood count, coagulation tests, and biochemistry analysis.

CXR of each study patient was acquired from the data storage platform and analyzed by two radiologists (VO and JN with 5 and 30 years of experience, respectively). Radiologists were unaware of patient clinical data, and only performed the images review process and scoring. In case of inter-reader discrepancies, they came to conclusions by consensus. Evaluation of CXR was performed using Brixia score, which detects lung damage by means of lung division into six sections, in which each section can achieve score of 0, 1, 2 or 3, while total score can go from 0 to 18. Greater score values are interpreted as more severe lung involvement [18]. We used the classification of patients into four groups based on the Brixia score. The first category was defined as normal and had a Brixia score of 0. The second category was defined as mild, with a Brixia score from 1 to 6. The third category was moderate, and had a Brixia score from 7 to 12, while fourth and last group was defined as severe and had a Brixia score from 13 to 18, according to the literature [19].

Variables investigated in the study were: demographic factors (age and gender), clinical presentation (based on oxygen blood saturation: mild SpO2 ≥ 94% and severe SpO2 < 94% on room air), laboratory parameters (red blood cell count, white blood cell count, lymphocyte count, platelet count, hemoglobin, C-reactive protein, procalcitonin, glucose, urea, creatinine, aspartate aminotransferase, alanine aminotransferase, gamma-glutamyl transferase, creatine kinase, creatine kinase-MB, lactate dehydrogenase, proBNP, troponin, albumin, D-dimer), CXR findings, vaccinal status and number of received doses, and length of hospitalization (in days). The dependent variables were treatment outcome and Brixia score. Treatment outcome was divided into two groups: patients who died during hospitalization (non-survivors) and those that were successfully treated (survivors).

All statistical analyses were performed using Statistical Program for Social Sciences (SPSS version 18). The data were analyzed by descriptive statistics. Measures of central tendency (mean and median) and measures of dispersion (standard deviation and range) were used for continuous variables depending on the normality of the data distribution. Normally distributed variables are presented as mean ± standard deviation, while median and range were used for non-normally distributed variables. Categorical variables were presented as frequencies and percentages (%). Normality of the data distribution in continuous variables was tested by Kolmogorov–Smirnov test and Shapiro–Wilk test, depending on which test’s assumptions were satisfied.

Spearman’s correlation coefficient was used to investigate correlations between Brixia score and laboratory parameters. Spearman’s correlation coefficient (ρ) < 0.4 was considered a weak correlation, ≥0.4 and <0.7 was considered a moderate correlation, and ≥0.7 was considered a strong correlation [20,21], while *p* values less than 0.05 were considered to indicate existence of statistical significance. Influence of potential predictors of Brixia score, including main laboratory parameters with significant moderate correlations with Brixia score, as well as age, gender, severe COVID-19, and COVID-19 vaccinal status, was evaluated by multiple linear regression using method “Enter”. Dichotomous categorical variables were coded with 0 and 1 (0 indicated absence of a qualitative attribute, while 1 indicated presence, except for gender where 0 indicated female gender, and 1 male gender). The statistical validity of the regression was checked by analysis of variance (F value) and percentage of the outcome (Brixia score) variability explained (R^2^). The influence of potential predictors on the outcome was assessed by their B coefficients in the regression equation, including 95% confidence intervals (CIs). A *p* value < 0.05 was considered statistically significant.

The differences in continuous variables between non-survivors and survivors were assessed by the independent group *t*-test when the data were normally distributed, or Mann–Whitney U test when the data were not normally distributed. Chi-squared (χ^2^) test or Fisher’s exact test were used to assess differences in categorical variables, depending on assumptions of which tests were satisfied. The differences were considered significant if the probability of null hypothesis was less than 0.05. In order to estimate the association between potential predictors and unfavorable outcome (death), crude and adjusted odds ratios (OR) with 95% CIs were calculated using univariate and multivariate logistic regression using method “Enter”. All variables which had significant crude OR in the univariate analysis were entered in the multivariate logistic regression analysis. If the 95% CI for the OR included the number 1 then the calculated OR was not considered to be statistically significant.

## 3. Results

This study included a total of 279 patients with confirmed COVID-19 with a median age of 62 years. There were more male patients (69.2%). A total of 33 patients died (11.8%) and 246 (88.2%) survived. Results also show that around two-thirds of patients had severe clinical presentation of COVID-19 pneumonia. In addition, a smaller portion of patients was vaccinated. Almost half of the patients had a mild Brixia score. Characteristics of the study population are shown in Table 1. 

Laboratory test results of the entire study population, survivors and non-survivors, are shown in the Table 2. 

**Figure 2:**
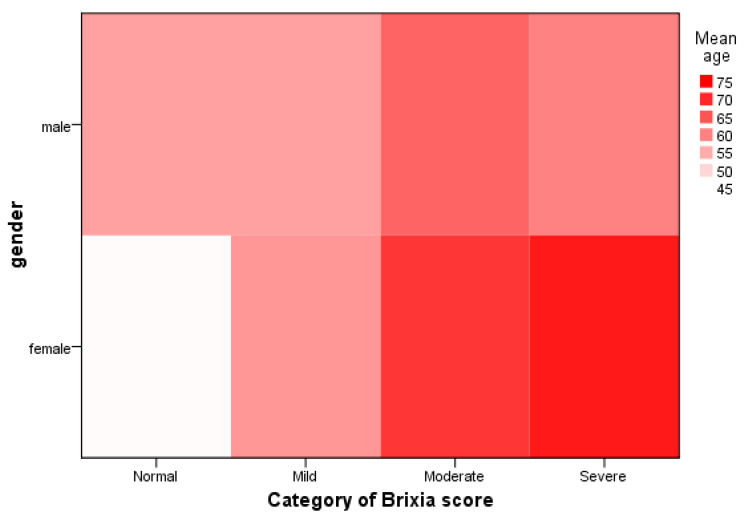
Heat map of Brixia score by gender and age of patients.

The median Brixia score in the study population was 5.0, and 49.8% of patients were categorized in mild Brixia score category. Only 34 patients (12.2%) received at least one dose of a COVID-19 vaccine. Brixia score was lower in vaccinated patients compared to unvaccinated patients (median [range] 4.0 [0.0–13.0] vs. 5.0 [0.0–17.0]), but the difference was not statistically significant (U = 3503.5, *p* = 0.131). We also evaluated Brixia score and its categories by gender and age of study patients and presented the results in Figure 2. The heat map demonstrates that the highest category of Brixia score was in a group of female patients who were oldest by age. Following this group was the group of patients of female sex and moderate Brixia score whose mean age was 65 years. The lowest values of Brixia score were in the group of female patients with a mean age of 45 years, while male patients in this age group had higher Brixia scores.

Brixia score had a significant moderately positive correlated with CRP and lactate dehydrogenase, while a significant moderate negative correlation was shown for albumin level. A significant weak positive correlation was found between Brixia score and white blood cell count, procalcitonin, glucose, urea, aspartate aminotransferase, alanine aminotransferase, gamma-glutamyl transferase, creatine kinase-MB, proBNP, troponin, and D-dimer, while a significant weak negative correlation was found between Brixia score and lymphocyte count. Significant values of Spearman’s correlation coefficients and the corresponding *p* values are shown in Table 3.

The results of the multiple linear regression analysis (R^2^ = 0.334, F = 19.424, *p* < 0.001) evaluating potential predictors of Brixia score are shown in Table 4. It was shown that male gender, severe COVID-19 and lactate dehydrogenase were significant positive predictors, while albumin level was a significant negative predictor of Brixia score.

Comparisons of differences in characteristics of survivors and non-survivors are shown in Table 5. Significant differences were found in age, severe COVID-19 frequency, Brixia score, white blood cell count, lymphocyte count, CRP, procalcitonin, glucose, urea, creatinine, aspartate aminotransferase, alanine aminotransferase, gamma-glutamyl transferase, creatine kinase-MB, lactate dehydrogenase, proBNP, troponin, albumin, and D-dimer.

The results of the multivariate logistic regression analysis (Cox and Snell R square 0.220, Nagelkerke R square 0.426, Hosmer–Lemeshow Chi square 1.600, df = 8, *p* = 0.991) after entering all the variables which had significant crude OR in the univariate analysis (age, severe COVID-19, Brixia score, white blood cell count, lymphocyte count, glucose, urea, creatinine, lactate dehydrogenase, and albumin) have shown that Brixia score was only significantly positively associated with unfavorable outcome (death) (adjusted OR 1.148, 95% CI 1.020–1.292, *p* = 0.022).

## 4. Discussion

In our study, we examined the CXR features, laboratory parameters, and treatment outcomes of hospitalized COVID-19 patients in a multicenter cohort. We investigated correlations between main laboratory parameters and treatment outcome with the Brixia score.

Our findings have shown that 16.8% of study patients had a normal Brixia score, while 49.8% were categorized as mild, 26.2% as moderate, and 7.2% as severe. The median Brixia score was 5.0. The only significant predictor of unfavorable outcome (death) was Brixia score (adjusted OR 1.148, 95% CI 1.020–1.292, *p* = 0.022). In addition, it was shown that male gender, severe COVID-19, and lactate dehydrogenase were significant positive predictors, while albumin level was a significant negative predictor of Brixia score. Only a small portion of our study population was vaccinated, 12.2% to be more exact.

A recently published study that investigated Brixia score in hospitalized COVID-19 patients has concluded that a higher score is connected to mortality, which was also observed in the previously published papers [6,7,22]. The study that examined CXR score as a clinical outcome predictor found that significant factors for the fatal outcome are patient age, SpO2, comorbidities, and Brixia score (*p* < 0.05) [23]. Our study also singled out Brixia score as the only significant independent mortality predictor. This is in concordance with our findings, and it may be very helpful for clinicians since the initial clinical picture may be in discordance with CXR findings and the initial Brixia score. Hence, the initial CXR may be beneficial in clinical decision-making to emphasize patients who are more likely to have a fatal outcome, which is especially of great benefit for doctors who work in critical care units.

Along with mortality, thrombotic complications and high values of D-dimer have been reported to correlate with Brixia score [5,8]. A significant positive correlation between the initial Brixia score and D-dimer value was found previously (r = 0.45, *p* < 0.000) [8]. Our study results are in agreement with this finding, also showing a positive correlation between these two parameters (ρ = 0.381, *p* < 0.001). The literature describes pathophysiological mechanisms of COVID-19 and thromboembolism, which includes endothelial dysfunction and micro-vascular thrombosis, and in this sense, this finding is applicable to utilizing initial Brixia score as a predictor for thromboembolic complications of COVID-19.

CRP was also identified as a factor with significant association with the initial Brixia score (r = 0.23, *p* <0.05) [8]. Our study also presented a similar finding with a stronger correlation compared to the previous study (ρ = 0.508, *p* < 0.001). It is expected to find higher values of CRP in patients with COVID-19 pneumonia because it is an inflammatory marker. Therefore, the mechanism of higher CRP values in patients with higher Brixia score is completely relatable and in concordance with the previously published literature.

Vaccinal status has been shown to be a significant factor for mortality prediction. One of the recent studies observed this only in univariate analyses, while multivariate logistic regression did not demonstrate this [23]. In studies with a higher rate of vaccinated patients (53.1%), Brixia score was higher in the unvaccinated COVID-19 patients (median, 5; interquartile range [IQR], 3–7) compared to the vaccinated ones (median, 1; IQR, 0–6) (*p* < 0.001), while normal CXR was found in 13% of the unvaccinated group and 36% of the vaccinated (*p* < 0.001) [24]. Others have presented that patients who were fully vaccinated had lower risk of admission into intensive care units (OR, 0.08 [95% CI: 0.09, 0.78; *p* = 0.02]) [25]. These data provide evidence of vaccine efficacy. Our study also examined the vaccination status of hospitalized COVID-19 patients, whereas Brixia score was higher in unvaccinated patients compared to vaccinated patients (5.0 [0.0–17.0] vs. 4.0 [0.0–13.0]), which is in concordance with similar studies. We have to note that in our study the vaccination rate was low (12.2%), which may influence the study results and could be a reason why statistical significance was not found. 

Our study had some limitations that need to be considered. Only hospitalized patients with more severe clinical symptoms were included in this study, or better say were hospitalized, while asymptomatic carriers and patients with mild clinical symptoms were treated at home and were not included in this study. Furthermore, other significant variables were not included in the study, such as the percentage of patients admitted to ICU, the need for mechanical ventilation, causes of death, complications, and comorbidities, since it was impossible to retrieve these data from our patients for our study database. It has to be noted that in the pandemic conditions, there was a surge of COVID-19 patients and so only patients with a severe clinical picture were admitted for hospital care. In addition, as previously mentioned, the number of vaccinated patients was low in our study population, our results showed good relatability with studies that investigated populations with higher vaccination rates.

However, our study results pointed out the importance of use of a scoring system in chest X-ray interpretation in conditions such as the COVID-19 pandemic. This should be of future reference for other epidemic/pandemic(s), but also in daily praxis, especially in patients in critical care, since comparison of the Brixia score in follow-up CXR can provide a much better orientation for the clinicians. Furthermore, different radiologists are interpreting CXR and in this way, results are more standardized.

## 5. Conclusions

Despite some limitations, our results provide important information about the factors influencing Brixia score and its usefulness in predicting unfavorable outcome (death) of COVID-19 patients. These findings have clinical relevance, especially in the epidemic circumstances and in lower income countries in general. Furthermore, it is important to put an emphasis on more severe clinical presentation and patients in critical care units during epidemic circumstances. In order to monitor these patients effectively and to achieve efficient patient management, CXR is a great tool due to its wide availability. In addition, the application of Brixia score is clear and simple, and benefits clinicians in daily praxis. 

We suggest more studies in this and similar topics in the future, especially with the use of more clinical, demographic, and laboratory markers, and the development of deep-learning prediction models which could be used in critical care daily routine.

## Figures and Tables

**Table 1:** Characteristics of the study population (*n* = 279).

Variable	Median (Range) or Number (%)
**Age (years)**	62 (18–98)
**Gender**	
**Male**	193 (69.2)
**Female**	86 (30.8)
**Length of hospitalization (days)**	12.0 (0.0–46.0)
**COVID-19 severity**	
**Mild**	85 (30.5)
**Severe**	194 (69.5)
**COVID-19 vaccination**	
**Yes**	34 (12.2)
**No**	245 (87.8)
**Number of received vaccine doses**	
**1**	13 (4.7)
**2**	17 (6.1)
**3**	4 (1.4)
**Brixia score**	5.0 (0.0–17.0)
**Brixia score category**	
**Normal**	47 (16.8)
**Mild**	139 (49.8)
**Moderate**	73 (26.2)
**Severe**	20 (7.2)
**Outcome**	
**Survived**	246 (88.2)
**Died**	33 (11.8)

Abbreviations: COVID-19—coronavirus disease 2019.

**Table 2:** Laboratory test results of the entire study population, survivors and non-survivors.

Variable	All Patients (*n* = 279)	Survivors (*n* = 246)	Non-Survivors (*n* = 33)
**Red blood cell count (10^12^/L)**	4.6 ± 0.6	4.6 ± 0.6	4.5 ± 0.6
**White blood cell count (10^9^/L)**	6.0 (1.8–29.2)	5.9 (1.8–29.2)	7.8 (2.1–19.6)
**Lymphocyte count (10^9^/L)**	0.9 (0.2–11.4)	0.9 (0.2–11.4)	0.7 (0.2–1.2)
**Platelet count (10^9^/L)**	191.0 (31.0–627.0)	190.5 (31.0–627.0)	194.0 (75.0–397.0)
**Hemoglobin (g/L)**	133.2 ± 16.1	133.6 ± 15.8	130.0 ± 18.1
**C-reactive protein (mg/L)**	71.3 (0.7–800.5)	62.4 (0.7–800.5)	95.7 (4.9–377.9)
**Procalcitonin (ng/mL)**	0.078 (0.010–21.190)	0.071 (0.010–21.190)	0.139 (0.020–3.740)
**Glucose (mmol/L)**	6.1 (2.7–24.2)	6.0 (3.3–24.2)	9.0 (2.7–19.5)
**Urea (mmol/L)**	6.0 (1.6–29.9)	5.4 (1.6–22.8)	9.8 (3.0–29.9)
**Creatinine (μmol/L)**	88.0 (46.0–1535.0)	88.0 (46.0–320.0)	88.0 (65.0–1535.0)
**Aspartate aminotransferase (IU/L)**	38.0 (13.0–1593.0)	36.0 (13.0–603.0)	56.0 (26.0–1593.0)
**Alanine aminotransferase (IU/L)**	34.0 (5.0–1220.0)	32.0 (5.0–721.0)	55.0 (20.0–1220.0)
**Gamma-glutamyl transferase (IU/L)**	37.0 (1.0–583.0)	35.0 (1.0–583.0)	43.0 (19.0–457.0)
**Creatine kinase (U/L)**	112.0 (22.0–3131.0)	109.5 (22.0–3131.0)	137.0 (28.0–2431.0)
**Creatine kinase-MB (U/L)**	12.0 (2.0–162.0)	12.0 (2.0–162.0)	14.0 (10.0–80.0)
**Lactate dehydrogenase (U/L)**	528.0 (105.0–5442.0)	512.0 (105.0–1461.0)	911.0 (336.0–5442.0)
**ProBNP (pg/mL)**	253.0 (5.0–35,000.0)	206.0 (5.0–35,000.0)	777.0 (33.0–35,000.0)
**Troponin (ng/mL)**	0.010 (0.001–46.484)	0.010 (0.001–46.484)	0.016 (0.003–1.270)
**Albumin (g/L)**	36.0 (18.0–51.0)	37.0 (18.0–51.0)	34.0 (23.0–40.0)
**D-dimer (mcg/L)**	0.7 (0.0–126.0)	0.6 (0.0–126.0)	1.0 (0.0–19.3)

Abbreviations: proBNP—pro–B-type natriuretic peptide. Results are presented as mean ± standard deviation or median (range) depending on the normality of data distribution.

**Table 3:** Significant correlations between Brixia score and laboratory parameters.

Laboratory Parameter	Spearman’s Correlation Coefficient (ρ)	*p*
C-reactive protein	0.508	<0.001 *
Lactate dehydrogenase	0.498	<0.001 *
Albumin	−0.482	<0.001 *
White blood cell count	0.236	<0.001 *
Procalcitonin	0.362	<0.001 *
Glucose	0.242	<0.001 *
Urea	0.211	<0.001 *
Aspartate aminotransferase	0.289	<0.001 *
Alanine aminotransferase	0.176	0.003 *
Gamma-glutamyl transferase	0.266	<0.001 *
Creatine kinase-MB	0.187	0.002 *
proBNP	0.374	<0.001 *
Troponin	0.208	<0.001 *
D-dimer	0.381	<0.001 *
Lymphocyte count	−0.256	<0.001 *

Abbreviations: proBNP—pro–B-type natriuretic peptide, *p*–statistical significance, *—statistically significant.

**Table 4:** Results of multiple linear regression analysis evaluating potential predictors of Brixia score.

Variable	B	95% CI	*p*
**Constant**	9.369	4.922; 13.816	<0.001 *
**Age**	0.003	−0.025; 0.031	0.833
**Male gender**	0.903	0.016; 1.790	0.046 *
**COVID-19 vaccinated**	−0.865	−2.101; 0.371	0.169
**Severe COVID-19**	1.970	0.989; 2.951	<0.001 *
**C-reactive protein**	0.003	−0.002; 0.008	0.312
**Lactate dehydrogenase**	0.002	0.001; 0.003	<0.001 *
**Albumin**	−0.211	−0.302; −0.120	<0.001 *

Abbreviations: COVID-19—coronavirus disease 2019, B—unstandardized coefficient, CI—confidence interval, *p*—statistical significance, *—statistically significant.

**Table 5:** Comparisons of differences in characteristics of survivors and non-survivors.

Variable	Survivors (*n* = 246)	Non-Survivors (*n* = 33)	Test Value	*p*	Crude Odds Ratios with 95 % Confidence Intervals for Unfavorable Outcome (Death)	*p*
**Age (years)**	62.0 (18.0–89.0)	68.0 (26.0–98.0)	U = 2921.0	0.009 *	1.036 (1.009–1.063)	0.009 *
**Male gender**	173 (70.3)	20 (60.6)	χ^2^ = 0.873	0.350	1.540 (0.728–3.261)	0.259
**Length of hospitalization (days)**	12.0 (0.0–46.0)	11.0 (1.0–38.0)	U = 3633.0	0.327	–0.978 (0.926–1.034)	0.442
**Severe COVID-19**	162 (65.9)	32 (97.0)	χ^2^ = 11.870	0.001 *	16.593 (2.228–123.554)	0.006 *
**COVID-19 vaccinated**	30 (12.2)	4 (12.1)	N/A	1.000	0.993 (0.326–3.022)	0.990
**Brixia score**	4.0 (0.0–16.0)	9.0 (0.0–17.0)	U = 1771.5	<0.001 *	1.291 (1.174–1.419)	<0.001 *
**Red blood cell count (10^12^/L)**	4.6 ± 0.6	4.5 ± 0.6	*t* = −0.609	0.543	0.832 (0.461–1.502)	0.832
**White blood cell count (10^9^/L)**	5.9 (1.8–29.2)	7.8 (2.1–19.6)	U = 3087.0	0.026 *	1.141 (1.045–1.245)	0.003 *
**Lymphocyte count (10^9^/L)**	0.9 (0.2–11.4)	0.7 (0.2–1.2)	U = 2155.5	<0.001 *	0.070 (0.018–0.267)	<0.001 *
**Platelet count (10^9^/L)**	190.5 (31.0–627.0)	194.0 (75.0–397.0)	U = 3750.0	0.478	0.998 (0.993–1.002)	0.334
**Hemoglobin (g/L)**	133.6 ± 15.8	130.0 ± 18.1	*t* = −1.198	0.232	0.987 (0.965–1.009)	0.232
**C-reactive protein (mg/L)**	62.4 (0.7–800.5)	95.7 (4.9–377.9)	U = 2991.5	0.014 *	1.003 (1.000–1.007)	0.050
**Procalcitonin (ng/mL)**	0.071 (0.010–21.190)	0.139 (0.020–3.740)	U = 2741.5	0.002 *	1.062 (0.891–1.267)	0.501
**Glucose (mmol/L)**	6.0 (3.3–24.2)	9.0 (2.7–19.5)	U = 2864.5	0.006 *	1.133 (1.043–1.232)	0.003 *
**Urea (mmol/L)**	5.4 (1.6–22.8)	9.8 (3.0–29.9)	U = 1892.0	<0.001*	1.205 (1.107–1.312)	<0.001 *
**Creatinine (μmol/L)**	88.0 (46.0–320.0)	88.0 (65.0–1535.0)	U = 3134.0	0.034 *	1.011 (1.002–1.021)	0.021 *
**Aspartate aminotransferase (IU/L)**	36.0 (13.0–603.0)	56.0 (26.0–1593.0)	U = 2293.0	<0.001 *	1.004 (0.999–1.010)	0.121
**Alanine aminotransferase (IU/L)**	32.0 (5.0–721.0)	55.0 (20.0–1220.0)	U = 2686.0	0.002 *	1.003 (1.000–1.006)	0.065
**Gamma-glutamyl transferase (IU/L)**	35.0 (1.0–583.0)	43.0 (19.0–457.0)	U = 3039.0	0.019 *	1.003 (0.999–1.007)	0.143
**Creatine kinase (U/L)**	109.5 (22.0–3131.0)	137.0 (28.0–2431.0)	U = 3823.5	0.588	1.000 (0.999–1.001)	0.741
**Creatine kinase-MB (U/L)**	12.0 (2.0–162.0)	14.0 (10.0–80.0)	U = 2777.0	0.003 *	1.018 (0.994–1.042)	0.146
**Lactate dehydrogenase (U/L)**	512.0 (105.0–1461.0)	911.0 (336.0–5442.0)	U = 1573.0	<0.001 *	1.004 (1.002–1.005)	<0.001 *
**ProBNP (pg/mL)**	206.0 (5.0–35,000.0)	777.0 (33.0–35,000.0)	U = 2260.5	<0.001 *	1.000 (1.000–1.000)	0.044
**Troponin (ng/mL)**	0.010 (0.001–46.484)	0.016 (0.003–1.270)	U = 2019.5	<0.001 *	0.978 (0.799–1.197)	0.828
**Albumin (g/L)**	37.0 (18.0–51.0)	34.0 (23.0–40.0)	U = 2093.0	<0.001 *	0.860 (0.799–0.925)	<0.001 *
**D-dimer (mcg/L)**	0.6 (0.0–126.0)	1.0 (0.0–19.3)	U = 2522.0	<0.001 *	1.008 (0.974–1.043)	0.647

Abbreviations: COVID-19—coronavirus disease 2019, proBNP—pro–B-type natriuretic peptide, U—Mann–Whitney U test value, *t*–independent group *t*-test value, χ^2^—χ^2^ test value, N/A—test value not applicable for Fisher’s exact test; *p*—significance of null hypothesis *—Statistically significant (note: for crude odds ratio both *p* < 0.05 and 95% confidence interval not including the value of 1). Continuous variables are presented as mean ± standard deviation or median (range) depending on the normality of data distribution. Categorical variables are presented as number (%).

## Data Availability

The datasets used and analyzed during the current study are made available from the corresponding author on reasonable request.
